# Effect of Drying Temperature on Sensory Quality, Flavor Components, and Bioactivity of Lichuan Black Tea Processed by Echa No. 10

**DOI:** 10.3390/molecules30020361

**Published:** 2025-01-17

**Authors:** Dan Su, Junyu Zhu, Yuchuan Li, Muxue Qin, Zhendong Lei, Jingtao Zhou, Zhi Yu, Yuqiong Chen, De Zhang, Dejiang Ni

**Affiliations:** National Key Laboratory for Germplasm Innovation & Utilization of Horticultural Crops, Huazhong Agricultural University, Wuhan 430070, Chinachenyq@mail.hzau.edu.cn (Y.C.);

**Keywords:** congou black tea, drying, sensory evaluation, aroma components, antioxidant property, enzyme activity

## Abstract

Lichuan black tea (LBT) is a well-known congou black tea in China, but there is relatively little research on its processing technology. Echa No. 10 is the main tea tree variety for producing LBT. This study investigated the sensory quality, flavor components, and bioactivity of Echa No. 10 Lichuan black tea (LBT) at different drying temperatures (70, 80, 90, 100, 110, 120, and 130 °C). During 80–120 °C, increasing the drying temperature enabled a higher sweet aroma concentration and enhanced the sweetness in the taste, in contrast to reducing the floral, fruity, and sweet aromas, and increasing the bitterness and astringency, at >120 °C. Additionally, with an increasing drying temperature, the contents of tea polyphenols and total catechins significantly decreased, with the theaflavins decreasing first and then increasing, and the alcohols, aldehydes, esters, and hydrocarbons increasing first and then decreasing. Meanwhile, compounds (including linalool, (Z)-linalool oxide (furanoid), (E)-linalool oxide (furanoid), cis-β-Ocimene, and methyl salicylate) contribute more to the floral and fruity aromas at <110 °C. Furthermore, low-temperature drying favors the antioxidant and inhibitory effects of the α-amylase, α-glucosidase, and glucose absorption activity. Both the tea quality and bioactivity results revealed 80–110 °C as the optimal drying temperature range for LBT.

## 1. Introduction

Black tea is a fully fermented beverage with a high consumer preference, owing to its distinctive flavor profile and remarkable physiological health benefits [1]. In China, black tea can be classified into souchong, congou, and broken black tea, with congou representing the primary category for the largest production and sales volume [2]. The fundamental processing steps of congou black tea encompass withering, rolling, fermenting, and drying, which collectively contribute to the development of the appearance, infusion color, aroma, and taste [3,4].

Drying is the final stage in black tea processing, serving to halt the fermentation by deactivating enzymes at high temperatures and to dehydrate the tea leaves. This process ensures the preservation and enhancement of the desirable qualities developed during fermentation [5,6]. Different drying methods can yield variations in the black tea quality. Lan et al. found that hot-air drying promoted the formation of sweet aroma substances in black tea, while strip drying, drum drying, and pan-fired drying were beneficial for developing fruity aromas [7]. Qu et al. demonstrated that, compared with the conventional hot-air drying, the black teas produced by microwave drying and halogen lamp–microwave drying showed superior color, taste, and aroma [8]. Wang et al. investigated the effects of five different drying methods (hot-air drying, drum drying, sun drying, vacuum drying, and freeze-drying) on the sensory quality of black tea, indicating that hot air-dried black tea, rather than vacuum-dried black tea, exhibited a superior sensory quality [9]. Lu et al. investigated the differences in the aroma of black tea under hot-air drying, sun drying, and pan-fired drying, showing that hot-air drying significantly enhanced the floral and roasted aroma of black tea [10]. The investigation by Ye et al. also indicated that, compared to drum drying, hot-air drying exhibited a superior antioxidant capacity [11]. The drying time and temperature also affect the quality of black tea. The investigation by Wang et al. discovered that the aroma profiles of black tea varied with the drying time, following a rise–fall trend with an increasing drying time, with drying at 85 °C for 45 min identified as a critical point influencing the aroma quality [12]. Su et al. demonstrated that increasing the drying temperature led to a gradual reduction in the levels of theaflavins, thearubigins, monosaccharides, and free amino acids, and a similar pattern was observed for the inhibitory effects on the α-glucosidase and α-amylase activity [13]. In China, different varieties of tea trees are used for congou black tea from different regions, but there is relatively little research on the processing technology of congou black tea with specific varieties.

Lichuan black tea (LBT), a well-known congou black tea in China, is produced from the fresh leaves of local cultivar Echa No. 10, the primary cultivar in Lichuan City, Enshi Autonomous Prefecture, Hubei, China. The tea is manufactured through the steps of withering, rolling, fermenting, and drying to attain its distinctive characteristics: a wiry and even appearance, a fairly red and bright infusion color, a mellow and fresh taste, a strong and long-lasting aroma, as well as red and bright infused leaves. Previous studies on LBT were primarily focused on industrial development and brand establishment [14], paying little attention to its processing technology and quality chemistry [15,16]. Currently, the research on its processing technology mainly revolves around variety optimization, withering methods, and fermentation degree. For example, Qin et al. compared the quality differences in Lichuan black teas made from nine tea tree varieties and found Echa No. 10, Baiya Qilan, and Meizhan were suitable for processing Lichuan black tea [17]. Zhou et al. investigated the flavor characteristics of Lichuan black tea with different fermentation degrees by using the flavor compound weighted network co-expression method [18]. However, few studies have been performed on specific process parameters, leading to variations among the different enterprises in the process parameters and significant differences in the quality of the final products, thus hindering effective brand-building and market promotion for LBT. To solve this problem, this study aimed to explore the optimal drying process parameters by analyzing the sensory qualities, flavor components, and bioactivity of LBT at different drying temperatures (70, 80, 90, 100, 110, 120, and 130 °C), which may provide useful information for LBT quality control.

## 2. Results

### 2.1. Effect of Drying Temperature on Sensory Quality of LBT

Table 1 shows the sensory evaluation results of the LBT at different drying temperatures, and Figure S1 illustrates the appearance of the dried tea, tea infusion color, and infused leaf color brightness. The scores for each quality factor of LBT followed a trend of increasing first and then decreasing with an increasing drying temperature. Specifically, higher drying temperatures (>120 °C) led to a reduction in the infusion brightness and a significant influence (*p* < 0.05) on the aroma and taste. During 70–120 °C, an increase in the drying temperature could enhance both the sweet aroma concentration and taste sweetness. However, at >120 °C, the floral and fruity aromas, as well as the sweet aroma concentration, decreased, coupled with an increase in the bitterness of the taste. Additionally, the infused leaf color brightness declined with an increasing drying temperature, leading to a corresponding decrease in the score.

**Figure 1:**
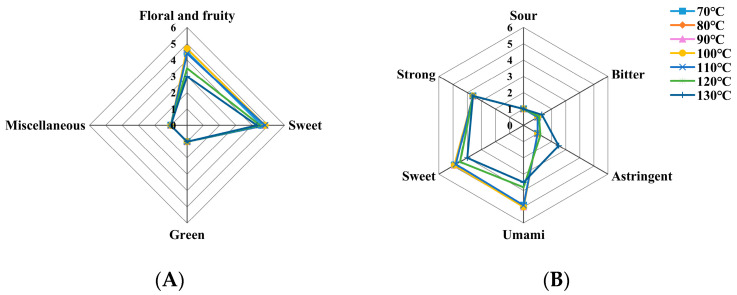
QDA radar chart of the aroma (**A**) and taste (**B**) of LBT at different drying temperatures.

Figure 1 illustrates the QDA scores of the aroma and taste factors of LBT at different drying temperatures. As shown in the radar chart, all of the samples exhibited the absence of a green or miscellaneous odor. Notably, the samples dried at 80, 90, and 100 °C had higher scores in terms of the floral and fruity aromas, as well as the sweet aroma, indicating that a suitable drying temperature range can enhance the aroma profile of black tea. In terms of the taste concentration, all of the samples showed no significant differences, but, compared to the other samples, the samples dried at 120 and 130 °C exhibited a higher level of bitterness and astringency, while exhibiting lower levels of sweetness and umami. This suggests that excessively high drying temperatures could exacerbate the tea’s bitterness and astringency, and compromise its overall quality. Based on the comprehensive evaluation results across various factors, the suitable drying temperature range can be concluded to be between 80 and 110 °C for Echa No. 10 LBT.

### 2.2. Effect of Drying Temperature on the Non-Volatile Components of LBT

During the drying process, various transformations occur in the tea constituents, including the thermal degradation of catechin and its oxidation products, the Maillard reaction involving amino acids and sugars, caramelization, as well as pigment degradation [19,20]. All of these reactions exert diverse effects on the sensory quality of the tea.

Table 2 shows the effect of different drying temperatures on the chemical composition of the LBT. The presence of soluble sugar in the tea infusion plays an important role in mitigating the bitter taste resulting from tea polyphenols and caffeine [21]. In the present study, the content of soluble sugar was significantly higher in the low-temperature-dried samples versus the high-temperature-dried samples, with 110 °C observed as an inflection point. This may explain the reason for the decline in the sweetness scores during the sensory evaluation from 110 °C onwards. Free amino acids are the primary contributors to the umami taste of tea [22], and are significant in the umami taste of tea infusions. Soluble sugars and free amino acids contribute to the sweet and umami taste of tea infusions, respectively, playing a crucial role in the flavor profile of black tea. However, under high-temperature conditions, they undergo the Maillard reaction and then are degraded by Strecker degradation to form aldehydes, pyrazines, pyrroles, and melanin [19]. This indicates that optimizing the content of soluble sugars and amino acids in tea can effectively enhance the quality of LBT, which can also be achieved through the controlled reduction in the drying temperature. The mellowness of the tea infusion can be partially characterized by the ratio of tea polyphenols to amino acids (T/A). Generally, a lower T/A indicates a higher level of umami and a mellower taste in the tea infusion. For LBT, the T/A decreased initially and then increased with a rising drying temperature, suggesting that a suitable drying temperature can enhance both the mellowness and umami levels in the tea infusion. Tea polysaccharide, an acidic polysaccharide or acidic glycoprotein combined with protein, exhibits diverse functionalities, such as antioxidation, blood sugar reduction, blood fat lowering, and immune enhancement. The content of tea polysaccharide increased with an increasing drying temperature due to the hydrolysis of the original pectin under high-temperature and acidic conditions, resulting in the formation of water-soluble pectin [23]. The properties of caffeine remained unchanged during drying, while the content of tea polyphenols significantly decreased due to the enhanced oxidation and degradation at higher temperatures [24]. Theaflavins, thearubigins, and theabrownin are crucial constituents related to the color, umami and mellow taste, as well as the astringency of black tea infusion [25]. With an increasing drying temperature, the levels of theaflavins and theabrownins followed a trend of decreasing first and then increasing at higher temperatures (120–130 °C). The content of thearubigins remained relatively stable between 70 and 120 °C, but significantly (*p* < 0.05) increased at 130 °C. There was a positive correlation between the theaflavin content and the black tea quality, while there was a negative correlation between the theabrownin content and black tea quality, suggesting that LBT can achieve a superior quality through low-temperature drying.

As the primary constituents of tea polyphenols, catechins exert a pivotal influence on the overall tea quality. As shown in Table 3, with an increasing drying temperature, the EGCG content remained relatively stable, the ECG content gradually decreased, and the EGC content exhibited a significant decrease and then became undetectable at 120 and 130 °C, in contrast to a substantial increase in the GC content from 120 °C onwards. Additionally, the C content witnessed a notable reduction between 70 and 100 °C, but with no significant changes at 110 °C. Moreover, with an increasing drying temperature, the amount of total catechins exhibited a significant decrease, which was consistent with the observed trend in the tea polyphenols. The above results indicated that the conversion of non-ester catechins could be more significantly influenced by the drying temperature.

The catechins undergo epimerization, hydrolysis, and oxidation/condensation reactions primarily under heating conditions, with their reaction degree varying with the drying temperature. Previous studies have demonstrated that EC and EGC are more heat-sensitive than other catechins, and their thermal instability can be mainly attributed to epimerization and oxidation/condensation reactions, respectively [26]. In the present study, an increase in the drying temperature led to a decrease in the contents of EGC, ECG, and C, indicating that higher temperatures exacerbated the intensity of epimerization and oxidation/condensation reactions. The significant rise in the GC content at 120 °C may be attributed to either epimerization or the degradation of other catechin monomers induced by the excessively high temperatures. Furthermore, the substantial increase in the gallic acid content also indicated that higher temperatures could promote the hydrolysis of catechins.

The changes in the monomer content and total theaflavins of the LBT at different drying temperatures are presented in Table 4. As the drying temperature increased, the contents of TF, TF3G, and total theaflavins exhibited a trend of increasing first and then decreasing, while the contents of TF3′G and TFDG gradually decreasing, indicating that excessively high drying temperatures (>120 °C) were not conducive to retaining the theaflavins. The heat sensitivity of theaflavins varies with their type, and prolonged exposure to high temperatures can cause the degradation of these compounds [8], which is consistent with our findings. Theaflavin is a crucial component for determining black tea quality, and an insufficient theaflavin content can result in a poor tea soup brightness. In the sensory evaluation, increasing the drying temperature was shown to reduce the score of the tea infusion brightness, which may be closely associated with decreased levels of theaflavins.

### 2.3. Effect of Drying Temperature on the Volatile Components of LBT

When dried in the temperature range of 70 to 110 °C, the LBT exhibited a sweet and fruity flavor. However, when dried at temperatures between 120 °C and 130 °C, the flavor became predominantly sweet. This indicated that the temperature could play a crucial role in the formation and transformation of volatile components in the tea. In order to investigate the aroma characteristics of the LBT under different drying temperatures, the volatile components in the tea samples dried at seven different temperatures were qualitatively and quantitatively analyzed using HS-SPME-GC-MS. According to Table S1, a total of 70 volatile compounds were identified, including 19 alcohols, 6 aldehydes, 5 ketones, 7 esters, 29 hydrocarbons, and 4 others. Notably, substances with relative contents exceeding 5 μg/kg included 3-hexen-1-ol, (Z)-linalool oxide (furanoid), (E)-linalool oxide (furanoid), linalool, phenylethyl alcohol, α-terpineol, cis-3,7-dimethyl-2,6-octadien-1-ol, geraniol, (Z)-citral, citral, cis-jasmone, methyl salicylate, 2-methyl-1-octene, β-myrcene, α-phellandrene, 1,3-cyclohexadiene, limonene, cis-β-ocimene, trans-β-ocimene, γ-terpinene, 1.3,4-dimethyl-2,4,6-octatriene, and neo-allo-ocimene.

The 70 common volatile compounds were subjected to principal component analysis (PCA), and, as shown in Figure S2, the samples dried at 70, 90, 100, and 110 °C were grouped together, while those dried at 120 and 130 °C formed another group. Notably, the samples dried at 80 °C exhibited significant differences from the other groups. These findings further supported the conclusion from the sensory evaluation results that the aroma score was higher in low-temperature-dried samples than in high-temperature-dried samples.

The volatile components in each sample, as indicated in Table S2, primarily comprised alcohols, hydrocarbons, esters, and aldehydes. With the increase in the drying temperature, the total amounts of alcohols, aldehydes, esters, hydrocarbons, and total volatile substances exhibited an uptrend first and then a downtrend. This suggested that high-temperature drying could cause the volatilization of numerous aroma compounds, particularly those with low boiling points. Notably, among all of the tested samples, the samples dried at 80 °C exhibited the highest concentration of aroma compounds. Furthermore, the loss of aroma compounds in tea was observed to gradually intensify with the drying temperatures beyond 80 °C.

The results of the volatile component OAV of the LBT at different drying temperatures are shown in Table 5. There are 11 OAV > 1 components, including β-myrcene (fruity), cis-β-ocimene (floral), trans-β-ocimene (sweet and herbal), (Z)-linalool oxide (furanoid) (woody and floral), (E)-linalool oxide (furanoid) (woody and floral), linalool (fruity and floral), nonanal (fruity), methyl salicylate (mint-like), geraniol (fruity and floral), α-cubebene (herbal), and cis-jasmone (floral) [27,28]. The OAV of these substances was generally higher in low-temperature-dried samples than in high-temperature-dried samples, which may explain the reason for the higher aroma concentration in the low-temperature-dried samples. Among the OAV > 1 components, cis-β-ocimene, linalool, and geraniol, with floral properties, exhibited higher OAVs, suggesting their significant contribution to the aroma formation of the LBT. This may explain the reason for the more pronounced floral and fruity characteristics in low-temperature-dried samples. Conversely, methyl salicylate, with a fresh and minty fragrance, exhibited an OAV of less than 1 in samples dried at 120 and 130 °C, which could account for the relatively simple aroma profile of the LBT dried at a high temperature.

**Figure 2:**
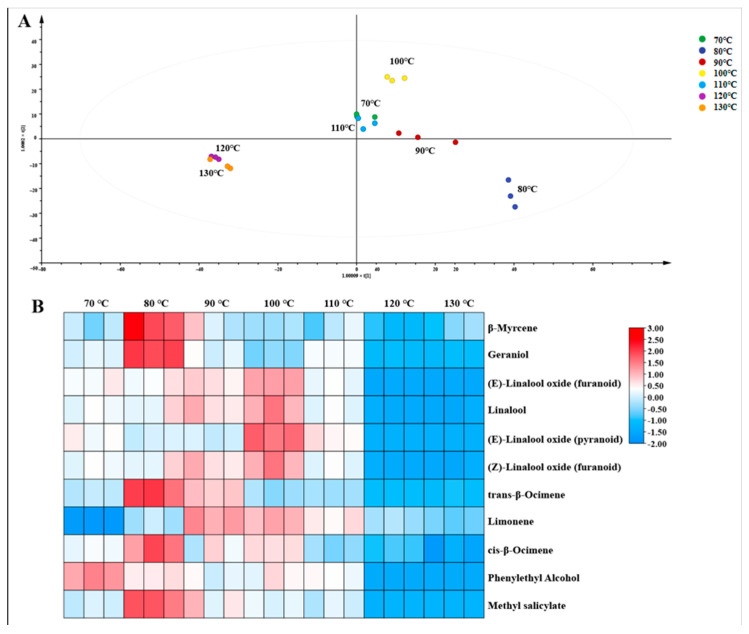
OPLS-DA score plot (**A**) and heat map (**B**) of the differential volatile components of the LBT at different drying temperatures.

Eleven differential volatile components (*p* < 0.05 and VIP > 1) were identified through the OPLS-DA (Figure 2A), including β-myrcene, cis-β-ocimene, trans-β-ocimene, (Z)-linalool oxide (furanoid), (E)-linalool oxide (furanoid), (E)-linalool oxide (pyranoid), linalool, methyl salicylate, geraniol, phenylethyl alcohol, and limonene. The thermographic analysis (Figure 2B) revealed that the contents of these compounds increased initially and then decreased with an increasing drying temperature, with most of them reaching the highest content in the temperature range of 80 to 100 °C. The joint analysis of eleven differential volatile components and eleven aroma compounds with an OAV > 1 identified eight key differential aroma components, and their correlations with aroma indices and aroma scores were further examined. As depicted in Figure S3, linalool, (Z)-linalool oxide (furanoid), (E)-linalool oxide (furanoid), cis-β-ocimene, and methyl salicylate exhibited significant positive correlations with a sweet aroma, floral aroma, and the overall aroma score. Linalool and its oxides have been established as crucial contributors to the floral and fruity aroma of black tea [29], while cis-β-ocimene is known to impart a fruity scent [30]. This may explain the reason for the higher sensory evaluation rates of floral and fruity aromas in samples dried at low temperatures than those at high temperatures.

### 2.4. Effect of Drying Temperature on the Bioactivity of LBT

Table 6 demonstrates the DPPH radical scavenging activity and α-amylase, α-glucosidase, and glucose uptake inhibitory activities of the LBT at different drying temperatures. The IC_50_ of the DPPH radical scavenging activity reached its minimum at 90 °C, significantly differing from the other treatments. The IC_50_ of the α-amylase inhibitory activity was significantly higher in the samples dried at high temperatures (100–130 °C) compared to those dried at low temperatures (70–90 °C). The IC_50_ of the α-glucosidase inhibitory activity increased with a rising drying temperature until reaching a maximum at 120 °C. The variation in the glucose transport inhibition rate with a changing drying temperature was not evident, with the IC_50_ peaking at 70 °C but showing no significant difference at 90 °C. Overall, maintaining the biological activity of the tea could be more favorable when the drying temperatures are kept below 110 °C.

The composition of the bioactive compounds in tea can be altered by the drying temperature, thereby impacting its biological activity. The relationship between non-volatile components and bioactivity indicators was further examined by a correlation analysis. As shown in Figure S4, the IC_50_ of the DPPH radical scavenging activity exhibited a negative correlation with C. Additionally, the IC_50_ of the α-amylase activity showed a negative correlation with the tea polyphenols, soluble sugars, free amino acids, TF3′G, TFDG, EGC, and C, but a positive correlation with gallic acid. Moreover, the IC_50_ of α-glucosidase demonstrated a negative correlation with the soluble sugar, TF3′G, TFDG, EGC, and total catechins, but a positive correlation with gallic acid. Furthermore, the inhibition rate of the glucose absorption was negatively correlated with gallic acid, but positively correlated with the tea polyphenols, soluble sugar, Tf3′G, EGC, C, ECG, and total catechins. Among the non-volatile components, phenolic compounds such as tea polyphenols, catechins, and theaflavins are widely acknowledged as the primary inhibitors of the enzyme activity and potent antioxidants [31]. The presence of hydroxyl groups and the aromatic structure in phenolic compounds are crucial contributors to their antioxidant and free radical scavenging effects [32]. Hydroxyl groups present in polyphenols can inhibit α-amylase activity by binding to its active sites. It is worth noting that theaflavins can exhibit distinct antioxidant effects [33], with the strongest inhibitory effect on the α-glucosidase activity for TFDG, followed by TF3G, TF3′G, and TF [34]. Gallic acid has been reported as one of the active antioxidant components in black tea, with a negative correlation between its content and antioxidant activity [35]. However, in the present study, a significant positive correlation was observed between gallic acid and the IC_50_ value of the α-amylase and α-glucosidase activity. Furthermore, black tea polysaccharide has been reported to exhibit a certain antioxidant capacity and a strong inhibitory effect on α-glucosidase [36]. However, in this study, no significant correlation was observed between the black tea polysaccharide and the biological activity of the tested samples. Collectively, the decrease in the catechin and theaflavin content, along with an increase in the gallic acid content, may be attributed to the decline in the antioxidant and hypoglycemic activities of the LBT with increasing drying temperatures. This suggests that reducing the drying temperature appropriately is advantageous for preserving the biological activities of LBT.

## 3. Materials and Methods

### 3.1. Chemicals and Reagents

Foline-phenol, methanol, sodium carbonate, ethyl acetate, n-butanol, ethanol, sodium bicarbonate, oxalic acid, aluminum trichloride, ninhydrin, anthrone, concentrated sulfuric acid, potassium dihydrogen phosphate, disodium hydrogen phosphate, stannous chloride, anhydrous sodium sulfate, disodium EDTA, ascorbic acid, iodine, and potassium iodide were purchased from Sinopharm Chemical Reagent Co., Ltd. (Shanghai, China); acetonitrile and glacial acetic acid were purchased from Aladdin biochemical technology Co., Ltd. (Shanghai, China); ethyl decanoate was purchased from Macklin biochemical technology Co., Ltd. (Shanghai, China); α-glucosidase, 4-nitrophenyl-α-d-glucopyranoside (pNPG), α-amylase, 2,2-diphenyl-1-picrylhydrazyl (DPPH), and starch were purchased from Yuanye bio-technology Co., Ltd. (Shanghai, China); the glucose content detection kit was from Solarbio technology Co., Ltd. (Beijing, China); the Caco-2 cell line was from Procell biotechnology Co., Ltd. (Wuhan, China); Dulbecco’s modified eagle medium (DMEM) and fetal bovine serum (FBS) were from Gibco (Waltham, MA, USA); the penicillin–streptomycin was from Labgic technology Co., Ltd. (Beijing, China); the nonessential amino acids were from Biological Industries (Israel), the L-glutamine was from Biofroxx (Einhausen, Germany), and the mycoplasma prevention reagent was from Yeasen biotechnology Co., Ltd. (Shanghai, China). All of the chemicals and reagents used in this study are of analytical grade.

### 3.2. Tea Sample Processing

**Figure 3:**
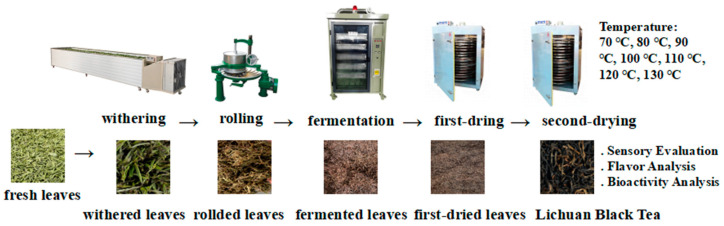
The process and experimental flowchart of Lichuan black tea.

The tea cultivar was Camellia sinensis cv. Echa No. 10, and the fresh leaf with a single bud proportion exceeding 90% was picked from the base of Lichuan Xingdoushan Black Tea Co., Ltd. (Hubei, China). Specifically, the fresh leaves were mixed well and spread evenly for withering in a 6CWD-200 withering tank (Zhejiang Green Peak Machinery Co., Ltd., Quzhou, China), with an initial cold-air blowing for 2 h, followed by hot-air blowing at 32 °C, until reaching a moisture content of 58%. After full rewetting, the rolling of the leaves was performed on a 6CR-55 rolling machine (Zhejiang Green Peak Machinery Co., Ltd., Quzhou, China) for 2 h in the sequence of light pressure, heavy pressure, and light pressure. After rolling, the leaves were fermented for 4 h at 32 °C and 95% humidity in a 6CFJ-400 fermenting machine (Zhejiang Green Peak Machinery Co., Ltd., Quzhou, China). After the fermentation, the leaves were first dried at 110 °C for 10 min in a 6CHZ-9B dryer (Fujian Jiayou Tea Machinery Intelligent Science and Technology Co., Ltd., Quanzhou, China) and then cooled to become soft at room temperature for about 1–2 h. After softening, the leaves underwent a second drying. Seven different second-drying temperatures (70, 80, 90, 100, 110, 120, and 130 °C) were set, and the leaves were dried until reaching a 6.5–7% moisture content. The processing and experimental flow of Lichuan black tea is shown in Figure 3.

### 3.3. Sensory Evaluation

Based on the Chinese National Standard [37], each black tea was independently evaluated by professional tea tasters on a 100-point scale, with 25% for the appearance, 10% for the infusion color, 25% for the aroma, 30% for the taste, and 10% for the infused leaf. Specifically, representative tea samples (100–200 g) were placed in a tea evaluation tray, with the tea leaves gently turned to change their positions. First, the appearance was assessed through visual inspection and tactile examination. Next, a 3 g tea sample was taken into an evaluation cup, filled with 150 mL of boiling water, covered, and brewed for 5 min, followed by filtration at a consistent speed to separate the tea infusion while leaving the infused leaves at the bottom of the cup. Subsequently, the evaluation was performed in the order of infusion color, aroma, taste, and infused leaf. Meanwhile, quantitative descriptive analysis (QDA) was performed to assess the specific sensory attributes of the tea [38]. Specifically, the appearance indicators included the moistness, tightness, fineness, and tippy; the infusion color and infused leaf indicators were the redness, brightness, and evenness; the aroma indicators comprised of floral, fruity, sweet, green, and miscellaneous indicators; the taste indicators encompassed sour, bitter, astringent, umami, sweet, and concentrated. The score was categorized into five grades, from 1 to 5, where 1 represented none or weak, while 5 represented strong.

### 3.4. Analysis of Tea Quality Components

#### 3.4.1. Determination of Total Tea Polyphenols

Each tea sample (0.2 g) was mixed with a 70% methanol solution (5 mL), followed by extraction in a 70 °C water bath for 10 min. After cooling to room temperature, the mixture was centrifuged at 2739× *g* for 10 min to separate the lower sediment and supernatant. This process was repeated twice, and then the supernatants were pooled together, followed by dilution to a final volume of 100 mL using a 70% methanol solution as an extract. The content of tea polyphenols in the extract was determined using the Folin–Ciocalteu method [39].

#### 3.4.2. Determination of Tea Pigments

Each tea sample (3 g) was mixed with boiling water (125 mL) and then immersed in a boiling water bath for 10 min. After hot suction filtration, the extract was obtained by cooling it rapidly to room temperature. The theaflavins, thearubigins, and theabrownins were extracted using ethyl acetate, a sodium bicarbonate solution, and n-butanol, respectively, and their contents were determined through systematic analysis [20].

#### 3.4.3. Determination of Free Amino Acids

The determination of free amino acids was performed according to previous research [40]. Echa tea sample (0.5 g) was mixed with boiling water (75 mL) and then soaked in a boiling water bath for 45 min, and an extract was obtained after hot filtration, centrifugation (at 2739× *g* for 10 min), and dilution with distilled water to a final volume of 100 mL.

#### 3.4.4. Determination of Total Flavones

Each tea sample (1 g) was mixed with boiling water (40 mL) and then extracted in a boiling water bath for 30 min. Subsequently, the hot mixture was suction-filtered and cooled to a constant volume of 50 mL using distilled water, which was obtained as an extract. The content of total flavonoids was determined by aluminum trichloride colorimetry [41].

#### 3.4.5. Determination of Soluble Sugar

Each tea sample (0.5 g) was mixed with boiling water (75 mL), followed by a boiling water bath for 45 min. Subsequently, the mixture was filtered while still hot, followed by centrifugation at 2739× *g* for 10 min, and dilution to a final volume of 100 mL with distilled water. After mixing 1 mL of the diluted solution with 2 mL of distilled water to obtain the test solution, the content of soluble sugar was determined by anthrone–sulfuric acid colorimetry [42].

#### 3.4.6. Determination of Tea Polysaccharide

Each tea sample (1 g) was mixed with an 80% ethanol solution (40 mL), followed by refluxing in a 95 °C water bath for 1 h. Subsequently, the hot mixture was suction-filtered and the solvent was evaporated. Then, the filter residue was leached with 100 °C boiling water (100 mL) for 1 h, followed by hot filtration and centrifugation (at 2739× *g* for 10 min). The resulting supernatant was diluted to a final volume of 100 mL with distilled water to obtain an extract, and the content of tea polysaccharide was determined using anthrone–sulfuric acid colorimetry [43].

#### 3.4.7. HPLC Determination of Catechins, Caffeine, Gallic Acid, and Theaflavins

Catechins, caffeine, gallic acid, and theaflavins were detected by high-performance liquid chromatography (HPLC) (1260 Infinity, Agilent, Santa Clara, CA, USA). the extract was obtained according to the Chinese National Standard [44], diluted by 80% with a stable solution (prepared with ultra-pure water, containing 5% 10 mg/mL EDTA-2Na, 5% 10 mg/mL ascorbic acid, and 10% acetonitrile), and filtered through a 0.45 μm filter membrane to obtain the test solution.

The HPLC determination conditions of the catechin components, caffeine, and gallic acid were as follows: The Agilent TC-C18 column (250 mm × 4.6 mm × 5 μm); a 35 °C column temperature, a 0.7 mL/min flow rate, a 5 µL injection volume, a 278 nm detection wavelength, the mobile phase A of ultra-pure water containing 0.1% formic acid, and the mobile phase B of methanol containing 0.1% formic acid. The elution gradient of the catechins, caffeine, and gallic acid consisted of 100–80% A from 0–2 min, 80–75% A from 2–6 min, 75–70% A from 6–10 min, 70–75% A from 10–13 min, 75–70% A from 13–23 min, 70–75% A from 23–25 min, 75–80% A from 25–30 min, and 80–100% A from 30–35 min.

The HPLC determination conditions of the theaflavin components were as follows: the Agilent TC-C18 column (250 mm × 4.6 mm × 5 μm); a 35 °C column temperature, a 0.7 mL/min flow rate, a 278 nm detection wavelength, a 5 µL injection volume, the mobile phase A of 90 mL acetonitrile with 20 mL glacial acetic acid and 2 mL EDTA-2Na (10 mg/mL), with ultra-pure water fixed to 1000 mL; the mobile phase B of 800 mL acetonitrile with 20 mL glacial acetic acid and 2 mL EDTA-2Na (10 mg/mL). The elution gradient of the theaflavins consisted of 100% A from 0–10 min, 100–68% A from 10–25 min, and 68–100% A from 25–35 min.

### 3.5. Gas Chromatography–Mass Spectrometry (GC–MS) Analysis

The volatile components were detected using gas chromatography–mass spectrometer (GC-MS) with the Thermo MS DSQ II (Thermo Fisher Scientific, Waltham, MA, USA). Briefly, the volatile components were adsorbed by headspace solid-phase microextraction (HS-SPME), and PDMS/DVB extraction fiber (PDMS/DVB 65 µm) was first conditioned at 250 °C for 1 h at the GC inlet. The sample of crushed tea (1.0 g) was placed into a headspace vial (20 mL) and extracted with 5 mL of boiled supersaturated NaCl solution, followed by adding 10 µL of ethyl decanoate internal standard solution (prepared using anhydrous ethanol, at 7.5 µg/mL), placing the bottle in a 60 °C water bath for 60 min after sealing it promptly [45].

The chromatographic conditions were as follows: a 30 mm × 0.25 mm × 0.22 µm DB-5MS column; a 230 °C inlet temperature; high-purity (≥99.99%) helium carrier gas; a 1.0 mL/min column flow rate; an initial temperature at 45 °C and holding for 2 min, then 7 °C/min to 80 °C with no hold, 2 °C/min to 90 °C and holding for 2 min, 3 °C/min to 100 °C and holding for 2 min, 3 °C/min to 130 °C and holding for 2 min, 3 °C/min to 150 °C, and, finally, 10 °C/min to 230 °C and holding for 5 min; a 40 °C column chamber temperature; splitless injection mode. The mass spectrometry conditions were as follows: an ion source EI; an electron energy of 70 eV; an ion source temperature at 230 °C; a mass scan range of *m/z* 32–400. The volatile components were characterized by the mass spectra, retention index (RI), and the NIST 2014 database.

The internal standard semi-quantitative method was employed to quantify the volatile components, with the formula calculated as follows: content of volatile component = peak area of volatile component × content of internal standard/peak area of internal standard. The odor activity value (OAV) was calculated as the ratio of the content of a volatile component to the odor threshold of the component.

### 3.6. Bioactivity Analysis

#### 3.6.1. Preparation of Tea Extracts

The ground tea sample (10 g) was extracted with boiling water (200 mL) at 100 °C for 10 min. After centrifugation at 2739× *g* for 10 min, the supernatant was collected and the residue was extracted once more under the same conditions. The two supernatants were pooled and concentrated using a vacuum rotary evaporator (RE-52AA, Shanghai Yarong Biochemistry Instrument Factory, Shanghai, China) at 55 °C under reduced pressure, followed by freeze-drying (Cool safe 110-4, Labogene ScanVac, Lynge, Denmark).

#### 3.6.2. DPPH Radical Scavenging Assay

The method for the DPPH radical scavenging assay was performed as previously reported, with slight modifications [46]. Briefly, samples (1 mL) with varying concentrations (5, 10, 15, 20, 30, and 40 μg/mL) were mixed with 2 mL of 0.15 mmol/L DPPH solution dissolved in absolute ethanol. Next, the mixture was incubated at room temperature in the dark for 30 min before measuring its absorbance at 517 nm using a microplate reader (KB288175, BioTek, Shoreline, WA, USA).

#### 3.6.3. Inhibitory Assay of α-Glucosidase

Following Qu’s method, with slight modifications [34], samples (50 μL) with varying concentrations (5, 7.5, 10, 15, 20, and 40 μg/mL) were mixed with 100 μL of α-glucosidase (1 unit/mL) in PBS (0.1 mol/L, pH of 6.8) in a 96-well plate and incubated in a biochemical incubator (SPX-150BIII, Tianjin Taisite instrument Co., Ltd., Tianjin, China) at 37 °C for 10 min. The catalysis reaction was initiated by adding 50 μL of pNPG (2.5 mmol/L) to each well and incubated at 37 °C for 5 min. Finally, the absorbance was measured at 405 nm using a microplate reader.

#### 3.6.4. Inhibitory Assay of α-Amylase

Based on the previous study, with slight modifications [34], samples (50 μL) with varying concentrations (5, 10, 15, 20, 30, and 40 μg/mL) were mixed with 50 μL of a-amylase (0.01 mg/mL) in PBS (0.1 mol/L, pH 6.8) in a 5 mL centrifuge tube and incubated at 37 °C for 5 min. The catalysis reaction was initiated by adding 1 mL of starch solution (2.0 mg/mL) to each centrifuge tube and incubated at 37 °C for 15 min, and terminated by adding 1 mL of an iodine diluent (0.01 mol/L). Finally, the absorbance was measured at 660 nm using a microplate reader.

#### 3.6.5. Inhibition of Glucose Uptake Assay by Caco-2 Cell Monolayers

According to slightly modified Qu’s method [34], the Caco-2 cells were seeded at a density of 105 cells/cm^2^ in a 12-well transwell inserts and incubated for 15–21 days in a CO_2_ incubator. Monolayers with transepithelial electrical resistance (TEER) values > 400 Ω·cm^2^ were used for the glucose uptake assay. Prior to the assay, the apical side of the monolayer was incubated in 0.6 mL of tea extracts (5 mg/mL) dissolved in DMEM, while the basolateral side was incubated in 1.2 mL of PBS for 2 h at 37 °C. Aliquots (50 μL each) of the solutions were drawn from the basolateral side to determine the glucose concentration as instructed by the glucose assay kit.

### 3.7. Statistical Analysis

The data were analyzed using SPSS statistical software version 25.0, and Fisher’s Least Significant Difference (LSD) approach was used to analyze the differences between the means, where *p* < 0.05 was considered statistically significant. SIMCA 14.1 software was used for the principal component analysis (PCA) and orthogonal partial least squares discriminant analysis (OPLS-DA) was used for the content of volatile components, the calculation of variable importance in projection (VIP), and screening for differential volatile components at *p* < 0.05 and VIP > 1. TB tools version 2.056 was used for the heatmap analysis.

## 4. Conclusions

The flavor quality and biological activity of Echa No. 10. LBT were significantly influenced by the drying temperature. The sensory quality of the LBT dried at temperatures ranging from 80 °C to 110 °C exhibited superior characteristics, including a pleasant sweetness and exquisite abundance of floral and fruity aromas. Drying below 110 °C was found to be more favorable for preserving the antioxidant and hypoglycemic activities of the LBT. Based on the comprehensive quality and bioactivity assessments, the optimal drying temperature for the Echa No. 10 LBT was determined to be between 80 °C and 110 °C. In the future, molecular sensory genomics methods can be further used to explore the key aroma compounds that have a significant impact on the floral and fruity aromas in low-temperature-dried tea samples, and it is also necessary to further expand the biological activity research. This study provides useful information for LBT drying parameter optimization to improve its quality.

## Figures and Tables

**Table 1:** Effect of different drying temperatures on the sensory quality of Lichuan black tea.

Sample	Appearance (25%)	Infusion Color (10%)	Aroma (25%)	Taste (30%)	Infused Leaf (10%)	Total Score
70 °C	86.16 ± 0.06 ^c^	90.55 ± 0.07 ^b^	90.75 ± 0.35 ^c^	90.77 ± 0.25 ^c^	88.25 ± 0.35 ^a^	89.34 ± 0.20 ^c^
80 °C	86.56 ± 0.08 ^b^	91.00 ± 0.00 ^a^	92.05 ± 0.07 ^b^	92.20 ± 0.26 ^a^	88.25 ± 0.35 ^a^	90.24 ± 0.12 ^ab^
90 °C	86.88 ± 0.11 ^a^	91.00 ± 0.00 ^a^	92.55 ± 0.07 ^a^	92.43 ± 0.12 ^a^	87.15 ± 0.21 ^b^	90.40 ± 0.04 ^a^
100 °C	86.54 ± 0.03 ^b^	90.65 ± 0.21 ^b^	92.55 ± 0.07 ^a^	92.20 ± 0.26 ^a^	86.25 ± 0.35 ^bc^	90.12 ± 0.12 ^ab^
110 °C	86.63 ± 0.04 ^b^	91.00 ± 0.00 ^a^	92.15 ± 0.21 ^ab^	91.77 ± 0.25 ^b^	86.25 ± 0.35 ^c^	89.95 ± 0.16 ^b^
120 °C	86.07 ± 0.10 ^c^	91.00 ± 0.00 ^a^	89.25 ± 0.35 ^d^	88.77 ± 0.25 ^d^	85.65 ± 0.21 ^d^	88.13 ± 0.19 ^d^
130 °C	85.61 ± 0.07 ^d^	90.10 ± 0.14 ^c^	88.25 ± 0.35 ^e^	87.17 ± 0.29 ^e^	85.25 ± 0.35 ^d^	87.15 ± 0.23 ^e^

Different lowercase letters in the same column indicate significant differences at *p* < 0.05.

**Table 2:** Effect of different drying temperatures on the non-volatile components of LBT (mg/g).

Sample	70 °C	80 °C	90 °C	100 °C	110 °C	120 °C	130 °C
Tea polyphenols	150.40 ± 0.79 ^a^	145.73 ± 1.18 ^b^	142.33 ± 1.85 ^c^	140.27 ± 2.50 ^cd^	137.29 ± 2.18 ^d^	139.20 ± 1.28 ^cd^	137.76 ± 2.08 ^d^
Free amino acids	39.23 ± 0.78 ^a^	39.33 ± 0.90 ^a^	38.57 ± 0.60 ^ab^	38.12 ± 0.34 ^ab^	37.79 ± 0.53 ^bc^	36.64 ± 0.67 ^c^	36.77 ± 0.96 ^c^
T/A	3.84	3.71	3.69	3.68	3.63	3.80	3.75
Theaflavins	3.07 ± 0.17 ^a^	2.81 ± 0.11 ^bc^	2.77 ± 0.21 ^bc^	2.63 ± 0.01 ^cd^	2.41 ± 0.13 ^d^	2.74 ± 0.16 ^bc^	2.98 ± 0.12 ^ab^
Thearubigins	38.87 ± 1.07 ^b^	38.40 ± 1.74 ^b^	38.18 ± 0.56 ^b^	38.74 ± 0.38 ^b^	38.26 ± 0.43 ^b^	38.35 ± 1.40 ^b^	42.19 ± 1.94 ^a^
Theabrownins	79.24 ± 0.38 ^bc^	78.14 ± 0.96 ^c^	78.89 ± 1.13 ^bc^	76.64 ± 0.60 ^cd^	74.89 ± 1.27 ^d^	81.46 ± 3.41 ^ab^	82.90 ± 1.15 ^a^
Soluble sugar	32.29 ± 0.28 ^a^	32.10 ± 0.67 ^a^	31.39 ± 0.62 ^a^	31.69 ± 0.81 ^a^	28.06 ± 0.11 ^b^	28.17 ± 0.71 ^b^	28.89 ± 1.45 ^b^
Tea polysaccharide	8.38 ± 0.26 ^d^	9.09 ± 0.30 ^c^	9.57 ± 0.49 ^c^	9.56 ± 0.32 ^bc^	9.61 ± 0.26 ^bc^	10.00 ± 0.37 ^b^	10.71 ± 0.14 ^a^
Caffeine	45.35 ± 0.54 ^b^	46.97 ± 1.31 ^a^	45.39 ± 0.37 ^b^	45.78 ± 0.64 ^ab^	45.86 ± 0.40 ^ab^	45.65 ± 0.64 ^b^	45.56 ± 0.46 ^b^
Gallic acid	3.10 ± 0.07 ^e^	3.62 ± 0.08 ^d^	3.57 ± 0.25 ^d^	4.44 ± 0.05 ^c^	5.04 ± 0.04 ^b^	5.48 ± 0.36 ^ab^	5.81 ± 0.50 ^a^

T/A refers to the ratio of tea polyphenols to amino acids. Different lowercase letters in the same row indicate significant differences at *p* < 0.05.

**Table 3:** Effect of different drying temperatures on the content of catechin components in LBT (mg/g).

Sample	GC	EGC	C	EGCG	ECG	Total Catechins
70 °C	11.04 ± 0.63 ^c^	6.75 ± 0.27 ^a^	10.48 ± 0.43 ^b^	5.85 ± 0.22 ^a^	4.61 ± 0.25 ^a^	39.15 ± 2.58 ^a^
80 °C	10.95 ± 0.68 ^c^	5.84 ± 0.31 ^b^	9.25 ± 1.13 ^c^	5.77 ± 0.56 ^a^	4.4 ± 0.33 ^ab^	38.76 ± 2.39 ^a^
90 °C	11.36 ± 0.29 ^c^	5.77 ± 0.49 ^b^	10.3 ± 1.37 ^a^	5.73 ± 0.11 ^a^	4.41 ± 0.10 ^ab^	36.53 ± 2.19 ^ab^
100 °C	11.62 ± 0.53 ^c^	4.22 ± 0.29 ^c^	8.16 ± 0.25 ^d^	5.58 ± 0.28 ^a^	4.29 ± 0.23 ^ab^	33.87 ± 0.88 ^bc^
110 °C	11.62 ± 0.45 ^c^	3.32 ± 0.06 ^d^	8.17 ± 0.14 ^d^	5.54 ± 0.16 ^a^	4.39 ± 0.10 ^ab^	33.04 ± 0.84 ^cd^
120 °C	12.75 ± 0.36 ^b^	--	8.02 ± 0.19 ^d^	5.66 ± 0.31 ^a^	4.11 ± 0.10 ^b^	30.64 ± 0.96 ^d^
130 °C	14.1 ± 0.39 ^a^	--	8.01 ± 0.10 ^d^	5.76 ± 0.17 ^a^	4.14 ± 0.33 ^b^	31.85 ± 0.73 ^cd^

Different lowercase letters in the same column indicate significant differences at *p* < 0.05; “--”, not detected.

**Table 4:** Effect of different drying temperatures on the content of theaflavins in LBT (mg/g).

Sample	TF	TF3G	TF3′G	TFDG	Total Theaflavins
70 °C	0.91 ± 0.02 ^b^	0.84 ± 0.06 ^ab^	1.45 ± 0.06 ^a^	14.54 ± 0.65 ^ab^	17.81 ± 0.51 ^b^
80 °C	0.94 ± 0.12 ^b^	0.86 ± 0.06 ^ab^	1.41 ± 0.12 ^ab^	14.81 ± 1.61 ^a^	19.30 ± 0.13 ^a^
90 °C	0.95 ± 0.18 ^b^	0.88 ± 0.08 ^ab^	1.40 ± 0.04 ^ab^	14.32 ± 0.42 ^ab^	17.55 ± 0.64 ^b^
100 °C	1.20 ± 0.03 ^a^	0.95 ± 0.08 ^a^	1.38 ± 0.02 ^abc^	14.40 ± 0.29 ^ab^	17.86 ± 0.33 ^b^
110 °C	0.96 ± 0.08 ^b^	0.91 ± 0.09 ^a^	1.28 ± 0.04 ^cd^	13.78 ± 0.39 ^ab^	17.05 ± 0.60 ^bc^
120 °C	0.87 ± 0.02 ^b^	0.79 ± 0.05 ^b^	1.23 ± 0.02 ^d^	13.36 ± 0.20 ^b^	16.26 ± 0.20 ^c^
130 °C	0.86 ± 0.04 ^b^	0.80 ± 0.06 ^ab^	1.27 ± 0.06 ^bcd^	13.37 ± 1.15 ^ab^	16.31 ± 1.28 ^bc^

Different lowercase letters in the same column indicate significant differences at *p* < 0.05.

**Table 5:** OAVs for the volatile components in the LBT at different drying temperatures.

Volatile Components	Threshold μg/kg	OAV						
		70 °C	80 °C	90 °C	100 °C	110 °C	120 °C	130 °C
β-Myrcene	15	21.60	40.15	25.66	21.13	21.59	14.95	18.93
cis-β-Ocimene	10	13.84	17.43	13.91	14.99	12.00	10.77	8.89
trans-β-Ocimene	34	5.45	10.27	7.82	4.93	5.01	3.23	3.26
(Z)-Linalool oxide (furanoid)	190	1.11	1.17	1.36	1.58	1.09	0.28	0.27
(E)-Linalool oxide (furanoid)	190	2.00	2.02	2.21	2.66	1.87	0.43	0.42
Linalool	0.22	1688.39	2620.19	2417.25	1858.59	1911.51	1429.72	1520.22
Nonanal	1.1	5.34	10.97	6.31	0.98	2.68	2.67	2.12
Methyl salicylate	40	1.59	2.63	1.94	1.70	1.58	0.83	0.83
Geraniol	7.5	47.58	77.02	48.66	37.79	50.54	28.63	28.66
α-Cubebene	0.8	3.88	12.38	5.87	4.46	5.59	3.96	4.24
cis-Jasmone	7	0.59	0.95	0.62	0.69	1.11	0.40	0.53

**Table 6:** Scavenging effects on the DPPH radical and inhibitory effects on the α-amylase, α-glucosidase, and glucose absorption activity of the LBT at different drying temperatures.

Samples	DPPH IC_50_ (μg/mL)	α-Amylase IC_50_ (μg/mL)	α-Glucosidase IC_50_ (μg/mL)	Inhibition Rate of Glucose Absorption (%)
70 °C	20.71 ± 0.23 ^d^	12.14 ± 0.08 ^b^	9.39 ± 0.77 ^b^	52.91 ± 0.68 ^a^
80 °C	21.02 ± 0.18 ^cd^	12.38 ± 0.03 ^b^	9.18 ± 0.27 ^bc^	49.67 ± 0.14 ^bc^
90 °C	19.71 ± 0.13 ^e^	11.22 ± 0.35 ^b^	8.25 ± 0.07 ^c^	52.15 ± 0.41 ^ab^
100 °C	22.30 ± 0.30 ^a^	15.37 ± 0.76 ^a^	9.18 ± 0.11 ^bc^	49.47 ± 0.65 ^bc^
110 °C	21.93 ± 0.22 ^ab^	16.00 ± 0.69 ^a^	10.87 ± 0.19 ^a^	46.04 ± 1.22 ^d^
120 °C	21.44 ± 0.22 ^bc^	15.6 ± 0.33 ^a^	10.50 ± 0.38 ^a^	46.51 ± 2.16 ^d^
130 °C	22.29 ± 0.31 ^a^	16.45 ± 0.33 ^a^	11.25 ± 0.44 ^a^	47.76 ± 0.68 ^cd^

Different lowercase letters in the same column indicate significant differences at *p* < 0.05.

## Data Availability

Data are contained within the article and Supplementary Materials.

## Supplementary Materials

The following supporting information can be downloaded at: https://www.mdpi.com/article/10.3390/molecules30020361/s1, Figure S1: The effect of drying temperatures on the appearance, infusion color, and infused leaves of LBT; Figure S2: PCA score plot of volatile components in the LBT at different drying temperatures; Figure S3: Correlation analysis of the key differential aroma components and the aroma quality of the LBT at different drying temperatures; Figure S4: Correlation analysis of non-volatile components and bioactivity of the LBT at different drying temperatures; Table S1: Content of volatile components in the LBT at different drying temperatures; Table S2: The effect of drying temperatures on the content of volatile components in the LBT.
