# Supplementary material for: Effect of Drying Temperature on Sensory Quality, Flavor Components, and Bioactivity of Lichuan Black Tea Processed by Echa No. 10
Source: Molecules. 2025 Jan 17;30(2):361. doi: 10.3390/molecules30020361 (PMC11767396; doi:10.3390/molecules30020361)
Supplement: Supplementary file 1 [file molecules-30-00361-s001.zip › molecules-3368606-supplementary.pdf]

Table S1 Content of volatile components in LBT at different drying temperatures（μg/kg-DW）

No.	RT	RI	Compound Name	70℃	80℃	90℃	100℃	110℃	120℃	130℃
			**Alcohols**							
1	4.15	854	3-Hexen-1-ol	18.65±0.82c	8.39±1.30d	14.26±0.34e	12.74±0.44e	9.15±1.00a	2.28±0.20d	2.36±0.42b
2	9.23	1069	(Z)-Linalool oxide (furanoid)	211.13±15.33a	222.28±32.07c	259.14±27.49d	299.94±28.85d	206.78±14.36c	52.53±5.06c	50.93±9.47b
3	9.80	1085	(E)-Linalool oxide (furanoid)	379.86±24.27a	384.15±31.53c	420.36±28.58d	505.79±3.30d	355.69±15.79c	82.04±2.01c	79.79±2.81b
4	10.31	1098	Linalool	371.45±40.48b	576.44±17.99b	531.79±74.1c	408.89±48.52c	420.53±35.26bc	314.54±12.29a	334.45±26.72a
5	10.74	1109	Phenylethyl Alcohol	69.19±5.61bc	52.69±1.88bc	43.52±7.27d	49.25±9.52d	46.95±2.17a	4.71±0.16b	4.48±0.37c
6	12.68	1153	(Z)-3-Nonen-1-ol	2.68±0.03cd	6.8±0.65bc	4.4±0.66d	3.74±0.07d	3.94±0.18e	3.36±0.22a	3.35±0.12b
7	12.77	1155	(E,Z)-3,6-Nonadien-1-ol	4.42±0.06c	8.16±0.65d	6.72±0.12e	5.29±0.19e	4.26±0.05d	2.54±0.12a	2.39±0.24b
8	13.23	1164	trans-2-cis-6-Nonadien-1-ol	0.19±0.02c	0.31±0.01bc	0.37±0.03b	0.24±0.03a	0.28±0.01d	0.3±0.03b	0.38±0.03a
No.	RT	RI	Compound Name	70℃	80℃	90℃	100℃	110℃	120℃	130℃

9	13.45	1168	(E)-Linalool oxide (pyranoid)	164.28±17.25a	132.77±8.34b	131.10±11.18d	271.70±13.79d	174.72±13.45b	14.30±0.31c	14.68±1.48c
10	13.56	1171	1-Nonanol	0.06±0.01c	0.26±0.02c	0.16±0.02a	0.06±0.00a	0.06±0.00c	1.43±0.02b	1.44±0.26bc
11	14.7	1192	α-Terpineol	6.38±0.49bc	10.84±1.74b	6.63±0.97d	7.02±0.86d	8.57±0.70c	3.40±0.24a	3.80±0.58c
12	14.81	1194	Myrtenol	1.14±0.08d	3.36±0.49c	2.62±0.31cd	1.82±0.26cd	2.21±0.20e	1.93±0.08a	2.04±0.06b
13	15.8	1213	7-Methyl-3-methylene-6-octen-1-ol	4.56±0.67b	8.74±1.09b	4.65±0.82b	4.40±0.33b	4.45±0.23b	4.07±0.04a	4.93±0.89b
14	16.16	1220	cis-3,7-Dimethyl-2,6-octadien-1-ol	7.68±0.62c	16.80±0.36b	8.54±1.09f	8.60±0.27e	9.94±0.01d	5.82±0.34a	6.68±0.11c
15	16.45	1226	(3Z)-3,7-Dimethyl-3,6-octadien-1-ol	2.96±0.52d	6.25±0.86bc	3.89±0.49e	2.64±0.33e	3.73±0.50cd	1.64±0.10a	1.29±0.01b
16	17.65	1248	Geraniol	356.84±13.83d	577.63±13.16b	364.94±34.38e	283.43±14.61e	379.06±0.33c	214.70±2.57a	214.95±7.81bc
17	18.41	1261	Citrol	0.31±0.03a	1.18±0.11a	1.28±0.12c	1.15±0.09b	1.22±0.12c	0.36±0.05a	0.63±0.00a
18	33.64	1551	Nerolidol	0.56±0.05c	1.33±0.18a	0.66±0.11a	0.60±0.02a	1.69±0.21c	1.72±0.12b	1.79±0.20c
19	36.34	1610	1,10-Diepicubenol	0.43±0.06d	0.87±0.15b	0.69±0.12a	0.46±0.03a	0.83±0.14d	1.04±0.07b	1.07±0.01c
No.	RT	RI	Compound Name	70℃	80℃	90℃	100℃	110℃	120℃	130℃

			**Aldehydes**							
20	4.92	900	Heptanal	1.30±0.22a	2.14±0.27a	2.00±0.32a	1.89±0.32b	1.89±0.31b	1.93±0.31a	1.32±0.12a
21	10.46	1102	Nonanal	5.88±0.06e	12.07±0.59d	6.94±0.71d	1.08±0.08d	2.95±0.43c	2.94±0.37a	2.33±0.22b
22	13.99	1179	3,7-Dimethyl-3,6-octadienal	2.48±0.18de	5.20±0.32d	3.22±0.30e	1.65±0.08e	1.89±0.03c	1.36±0.11a	1.39±0.20b
23	15.29	1203	Decanal	2.22±0.32d	5.05±0.77cd	2.94±0.11bc	1.88±0.10bc	2.28±0.09cd	2.66±0.10a	2.59±0.25b
24	16.90	1234	(Z)-Citral	7.84±0.26c	18.20±3.15bc	9.23±0.95bc	5.75±1.06bc	8.14±0.62bc	7.56±0.40a	7.67±1.29b
25	18.66	1265	Citral	11.10±0.26cd	24.58±3.72de	14.36±0.13ef	9.77±0.34f	8.68±1.91c	7.01±0.30a	6.69±0.36b
			**Ketones**							
26	12.33	1146	Chrysanthenone	0.69±0.01c	1.82±0.09ef	1.46±0.09f	0.88±0.12e	0.39±0.03d	0.34±0.04a	0.45±0.07b
27	24.44	1371	7-epi-cis-Sesquisabinene hydrate	0.17±0.01de	0.27±0.02a	0.24±0.02c	0.23±0.02b	0.86±0.15e	0.36±0.04cd	0.52±0.08de
28	25.11	1383	cis-Jasmone	4.12±0.11b	6.64±0.79a	4.34±0.39c	4.81±0.58bc	7.78±1.60b	2.81±0.34a	3.70±0.64b
No.	RT	RI	Compound Name	70℃	80℃	90℃	100℃	110℃	120℃	130℃

No.	RT	RI	Compound Name	70°C	80°C	90°C	100°C	110°C	120°C	130°C
29	26.48	1408	α-Ionone	0.57±0.07c	0.82±0.00c	0.25±0.05d	0.39±0.05cd	0.39±0.01b	0.33±0.00a	0.38±0.02e
30	27.93	1437	Nerylacetone	0.29±0.04c	0.76±0.07c	0.58±0.06b	0.46±0.06b	0.42±0.03d	0.64±0.09a	0.65±0.05b
			**Esters**							
31	14.26	1184	(E)-3-Hexen-1-yl butyrate	4.30±0.28c	14.27±1.12c	10.07±0.30b	7.17±0.47b	7.19±0.75d	9.44±0.26a	9.50±0.42b
32	14.50	1188	Methyl salicylate	63.76±2.60c	105.07±4.66c	77.59±15.07d	67.92±2.12d	63.31±7.05c	33.07±0.92a	33.16±0.02b
33	16.56	1228	(3Z)-3-Hexen-1-yl 3-ethylbutanoate	2.00±0.24c	5.66±0.51c	3.86±0.02b	2.09±0.13b	2.50±0.20c	3.98±0.17a	3.94±0.74b
34	20.41	1293	Geranyl formate	1.13±0.00c	1.79±0.14c	1.02±0.11d	0.66±0.11d	0.73±0.12b	0.47±0.05a	0.51±0.05b
35	24.1	1365	Isobutyric acid 2-Ethyl-3-hydroxyhexyl ester	0.30±0.01c	0.35±0.05a	0.33±0.01c	0.26±0.03c	0.36±0.04b	0.23±0.03a	0.25±0.05ab
36	24.72	1376	(Z)-Hexanoic acid, 3-hexenyl ester	1.99±0.28d	8.09±1.10c	4.04±0.31bc	2.87±0.30b	4.18±0.68d	4.57±0.40a	5.35±0.34c
37	25.00	1381	Hexyl hexanoate	0.34±0.02c	0.53±0.05d	0.67±0.12ab	0.39±0.03a	0.23±0.05c	0.62±0.05b	0.66±0.04a

			**Hydrocarbons**							
38	4.34	866	2-Methyl-1-octene	8.55±0.11a	7.45±1.81d	9.17±0.44e	11.46±0.48e	7.28±1.17bc	2.25±0.39cd	1.90±0.13b
39	6.84	989	β-Myrcene	324.06±37.04bc	602.22±50.53bc	384.95±69.30d	316.89±7.57cd	323.78±60.70bc	224.23±16.98a	283.88±41.17b
40	7.3	1007	α-Phellandrene	19.59±1.33c	38.88±3.80c	30.37±1.20d	19.95±1.28d	19.56±1.04c	12.86±0.41a	12.23±1.42b
41	7.61	1018	1,3-Cyclohexadiene	21.73±0.71d	46.57±0.55c	40.51±0.85f	23.68±0.29f	25.67±0.77e	16.38±1.16a	16.86±0.51b
42	7.97	1030	Limonene	23.12±0.34a	43.82±2.32b	62.81±3.40c	60.18±2.41d	53.85±1.90e	43.01±1.26c	38.30±1.47a
43	8.08	1034	cis-β-Ocimene	138.44±2.44b	174.27±14.47c	139.07±19.20c	149.88±0.63d	119.95±5.40b	107.72±2.97a	88.91±9.12b
44	8.43	1045	trans-β-Ocimene	185.42±7.70d	349.27±21.38cd	265.97±9.15e	167.53±15.39e	170.49±3.71c	109.79±0.41a	110.92±8.69b
45	8.84	1058	γ-Terpinene	14.57±1.30c	27.84±1.63c	22.12±3.30d	12.79±0.31c	15.11±1.31c	9.75±0.75a	12.78±0.61b
46	9.59	1079	Cyclohexene	0.56±0.08b	1.83±0.13b	0.72±0.06c	0.73±0.08c	0.76±0.07c	0.47±0.06a	0.52±0.08b
47	11.19	1120	Cosmene	1.63±0.22bc	3.84±0.74bc	2.47±0.24de	2.11±0.26e	2.11±0.07cd	1.15±0.08a	1.01±0.16b
No.	RT	RI	Compound Name	70℃	80℃	90℃	100℃	110℃	120℃	130℃

48	11.51	1127	1.3,4-Dimethyl-2,4,6-octatriene	20.01±2.38d	41.63±0.37c	30.21±0.60e	20.37±0.11e	22.28±0.30d	11.69±0.59a	11.68±0.95b
49	12.06	1140	cis-Allocimene	10.65±0.16cd	30.77±1.24c	22.46±0.74e	12.41±0.51e	13.14±1.86d	8.01±1.47a	6.47±0.52b
50	19.16	1274	2,6,11-Trimethyldodecane	2.16±0.38b	2.95±0.51b	3.14±0.41c	2.22±0.29b	1.95±0.38b	1.34±0.17a	2.07±0.28a
51	22.85	1341	α-Cubebene	3.10±0.35c	9.90±1.05b	4.70±0.05c	3.57±0.00c	4.47±0.75c	3.17±0.20a	3.39±0.31b
52	24.25	1368	α-Copaene	1.18±0.07a	1.47±0.13ab	1.45±0.19d	1.78±0.06d	1.59±0.30c	0.85±0.09b	0.81±0.05b
53	25.76	1394	Tetradecane	1.62±0.04a	2.50±0.14b	2.09±0.03f	3.51±0.25f	2.87±0.02e	1.03±0.03c	1.05±0.14d
54	26.11	1401	(-)-α-Cedrene	0.36±0.04a	0.34±0.05a	0.34±0.06a	0.33±0.02a	0.33±0.02a	0.33±0.04a	0.33±0.02a
55	26.28	1404	(+)-Longifolene	0.45±0.08b	1.04±0.17b	0.7±0.01cd	0.75±0.03bc	0.70±0.14d	0.52±0.03a	0.65±0.04b
56	26.85	1416	γ-Elemene	0.78±0.12bc	1.50±0.27b	0.89±0.09bc	0.95±0.13bc	1.06±0.10c	0.94±0.13a	0.92±0.05bc
57	27.68	1433	β-Copaene	0.75±0.04d	2.30±0.02b	1.09±0.15bc	0.75±0.12bc	1.28±0.15d	1.13±0.10a	1.21±0.11c
58	28.13	1441	(E)-β-Famesene	0.74±0.06c	2.82±0.53b	1.24±0.19b	0.82±0.01b	1.54±0.18c	1.54±0.19a	1.52±0.11b
No.	RT	RI	Compound Name	70℃	80℃	90℃	100℃	110℃	120℃	130℃

59	28.88	1456	Cadina-3,5-diene	1.03±0.04b	4.29±0.62c	1.96±0.03b	1.63±0.22b	0.98±0.17c	1.84±0.17a	1.97±0.17b
60	29.08	1460	α-Elemene	0.36±0.04bc	0.77±0.01b	0.43±0.03cd	0.43±0.01cd	0.47±0.02d	0.39±0.04a	0.41±0.05bc
61	29.85	1474	γ-Muurolene	0.21±0.03d	1.62±0.01d	0.50±0.00c	0.54±0.00b	0.53±0.02e	0.68±0.11a	0.92±0.03d
62	30.34	1483	α-Amorphene	0.74±0.13c	1.57±0.22c	0.85±0.15b	0.67±0.09b	0.67±0.02bc	0.96±0.18a	0.91±0.07bc
63	31.34	1501	(+)-δ-Cadinene	3.58±0.04d	10.43±1.56b	6.21±0.19c	5.20±0.97cd	8.31±1.65e	6.69±0.61a	6.29±0.19cd
64	31.49	1504	(-)-Calamenene	1.16±0.09c	2.54±0.48d	1.81±0.17c	1.37±0.22b	0.86±0.11c	1.31±0.06a	1.69±0.06b
65	32.01	1516	Cubenene	1.03±0.00cd	2.51±0.47e	1.71±0.13c	1.15±0.08c	0.87±0.07de	1.34±0.10a	1.39±0.09b
66	32.38	1524	α-Calacorene	0.45±0.09e	0.63±0.12de	0.55±0.04b	0.34±0.04a	0.35±0.07cd	0.56±0.04ab	0.71±0.06bc
			**Others**							
67	19.97	1287	Thymol	0.92±0.15c	1.91±0.14a	1.50±0.02d	1.43±0.15c	2.18±0.19d	0.86±0.05b	1.40±0.16c
68	21.4	1312	Diosphenol	1.24±0.17bc	2.66±0.50bc	1.84±0.16b	1.64±0.12bc	1.55±0.14c	1.67±0.12a	1.58±0.19b
No.	RT	RI	Compound Name	70°C	80°C	90°C	100°C	110°C	120°C	130°C

69	30.91	1493	2,5-Di-tert-butylphenol	1.12±0.09cd	2.30±0.40ab	0.78±0.10b	0.88±0.14b	2.00±0.28c	1.81±0.29a	1.94±0.17d
70	20.21	1290	2,6,10,10-Tetramethyl-1-oxaspiro[4.5]dec-6-ene	0.24±0.02bc	0.71±0.10bc	0.41±0.04a	0.33±0.02b	0.33±0.04c	0.69±0.10a	0.41±0.06b

Note: Retention index is determined by using the homologous series of n-alkanes (C8 - C24); Data are presented as mean ± standard deviation (n ⩾ 3). Different lowercase letters in the same line indicate significant differences at $p<0.05$. RT, retention time; RI, retention index.

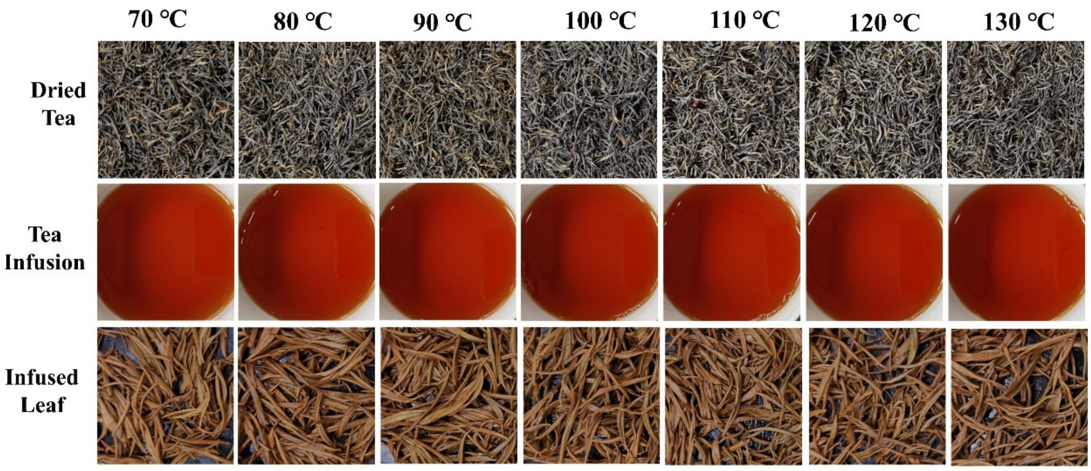

Figure S1 The effect of drying temperatures on the appearance, infusion color, and infused leaf of LBT

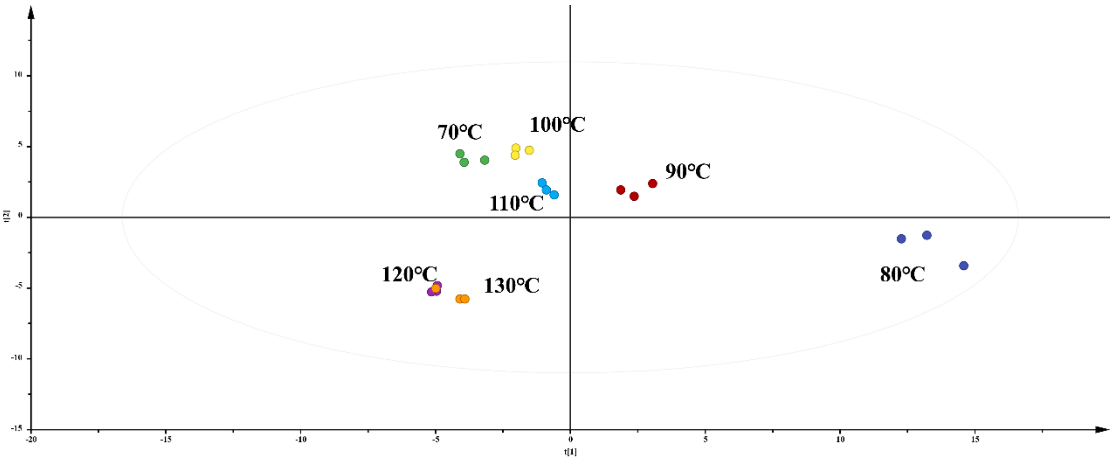

Figure S2 PCA score plot of volatile components in LBT at different drying temperatures

Table S2 The effect of drying temperatures on the content of volatile components in LBT（μg/kg-DW）

Sample	70℃	80℃	90℃	100℃	110℃	120℃	130℃
Alcohols	1602.75±68.96c	2019.26±51.83a	1805.73±150.55b	1867.76±84.60b	1634.07±66.86c	712.70±15.99d	731.43±34.61d
Aldehydes	30.82±0.44c	67.23±5.26a	38.69±1.05b	22.03±0.70e	25.84±1.54d	23.47±0.31de	21.98±1.10e
Ketones	5.85±0.02b	10.31±0.69a	6.87±0.38b	6.77±0.37b	9.84±1.67a	4.48±0.34c	5.69±0.69bc
Esters	73.83±2.11c	135.78±6.23a	97.59±10.04b	81.35±2.10c	78.50±5.38c	52.37±0.48d	53.37±1.33d
Hydrocarbons	788.01±32.85c	1417.57±63.45a	1040.47±67.59b	823.97±13.93c	802.79±59.16c	571.92±14.77d	611.67±51.62d
Others	3.53±0.12f	7.58±0.72a	4.53±0.27de	4.29±0.14e	6.06±0.13b	5.02±0.27cd	5.33±0.13c
Total amount	2504.78±81.67d	3657.74±27.28a	2993.88±226.12b	2806.17±70.52c	2557.10±62.53d	1369.96±30.60e	1429.49±86.26e

Note: Different lowercase letters in the same row indicate significant differences at the $p<0.05$.

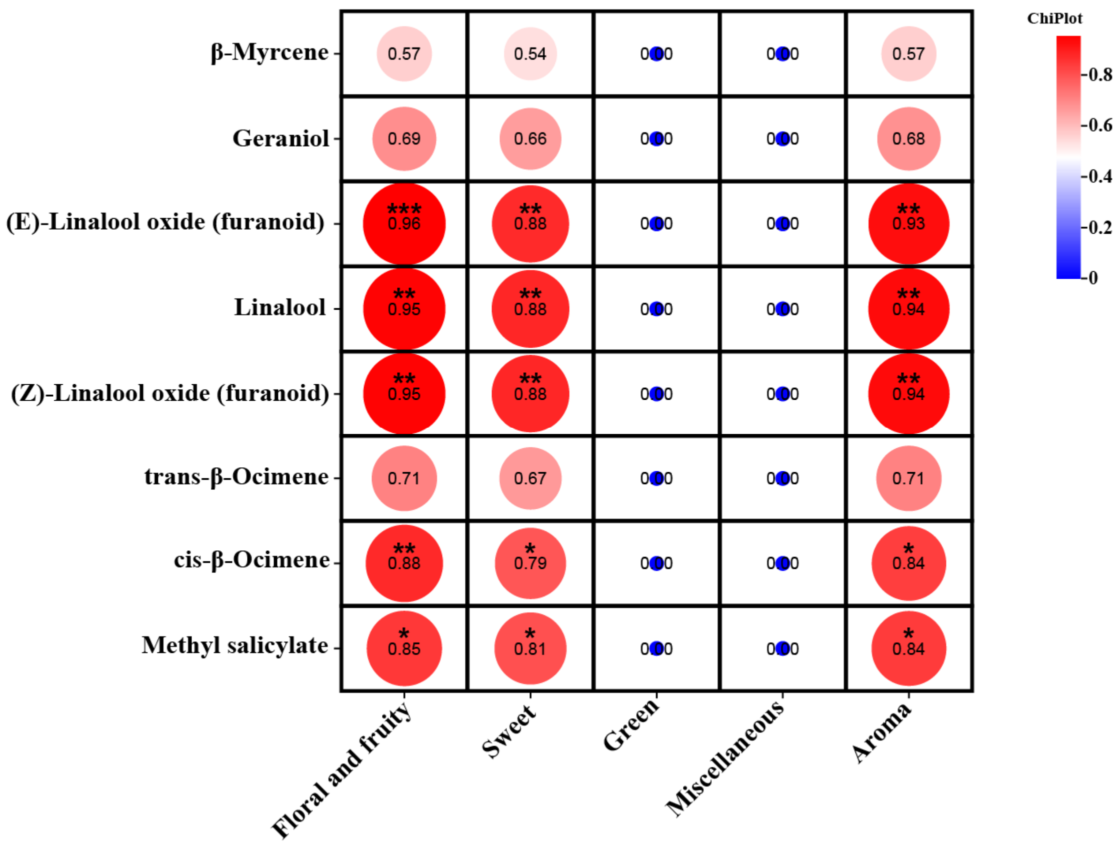

Figure S3 Correlation analysis of key differential aroma components and aroma quality of LBT at different drying temperatures

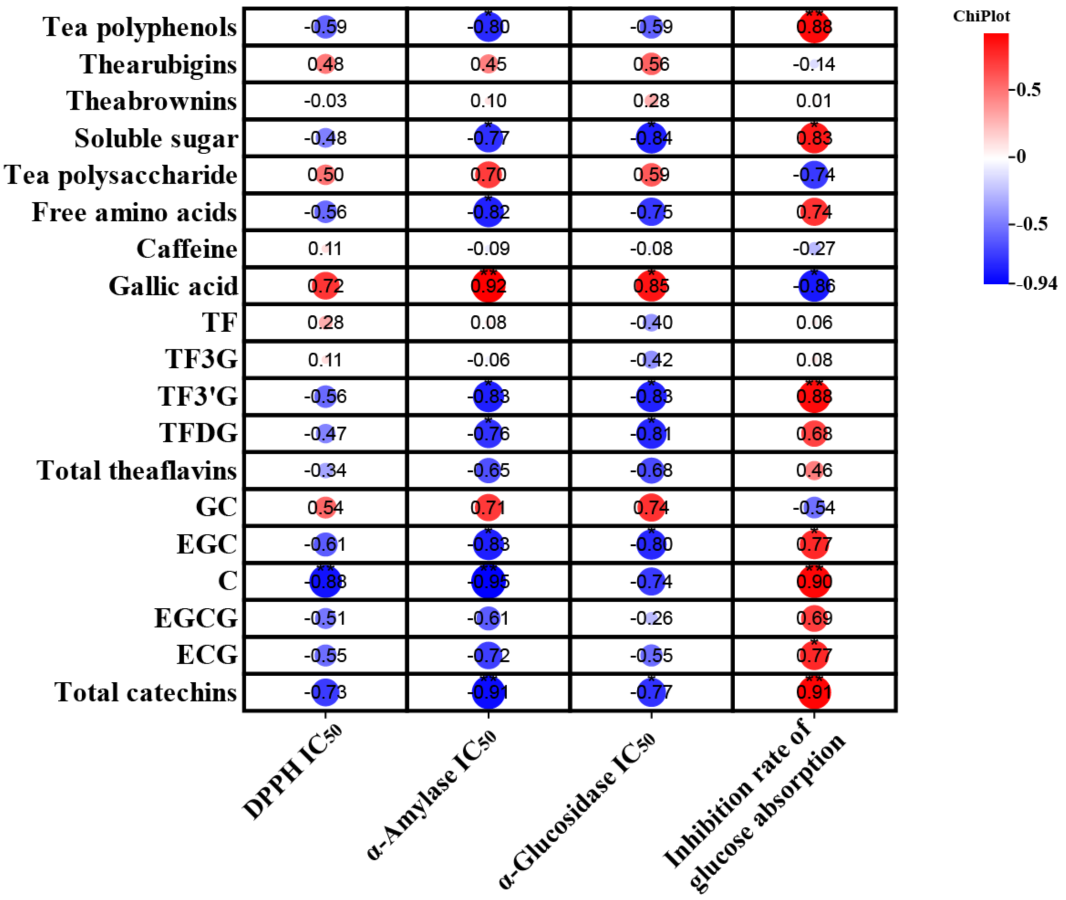

Figure S4 Correlation analysis of non-volatile components and bioactivity of LBT at different drying temperatures
